# CXCL1-CXCR2 axis inhibits melanogenesis through the suppression of the WNT/β-catenin pathway

**DOI:** 10.3389/fimmu.2026.1907373

**Published:** 2026-07-31

**Authors:** Yushan Zhang, Fan Zhang, Xiaoyuan Yu, Xiaojiao Zhao, Jing Chen, Jianjian Zhu, Liyang Kang

**Affiliations:** 1 Department of Dermatology, The Third Xiangya Hospital, Central South University, Changsha, China; 2 Department of Dermatology, Changde Hospital, Xiangya School of Medicine, Central South University, The First People’s Hospital of Changde City, Changde, China

**Keywords:** CXCL1, CXCR2, melanogenesis, pigmentary skin disorders, Wnt/β-catenin signaling pathway

## Abstract

Abnormal skin pigmentation is a common clinical dermatological issue, and its occurrence and development are closely associated with the inflammatory microenvironment. However, the interactive regulatory mechanisms between inflammatory factors and melanogenesis remain incompletely understood. In this study, through the analysis of transcriptomic data from melasma, psoriasis, acne, atopic dermatitis, and ultraviolet-irradiated skin tissues, we found that CXCL1 may negatively regulate melanogenesis. To validate this finding, we exogenously treated human primary melanocytes, MNT1 cells, and ex vivo human foreskin tissues with CXCL1. The results showed that CXCL1 reduced melanin content, tyrosinase activity, and the expression of key melanogenesis-related genes, including MITF, TYR, TYRP1, and DCT. Mechanistic studies revealed that CXCL1 exerts these inhibitory effects through its canonical receptor CXCR2. Notably, CXCL1 treatment significantly decreased both the expression level and nuclear translocation of β-catenin, a key effector molecule of the WNT signaling pathway, and this effect was effectively reversed by the specific CXCR2 inhibitor SB225002, suggesting that the CXCL1-CXCR2 axis negatively regulates melanogenesis by suppressing the WNT/β-catenin signaling pathway. In summary, this study elucidates a preliminary mechanism by which the CXCL1-CXCR2 axis negatively regulates melanogenesis through inhibition of the WNT/β-catenin signaling pathway, linking inflammatory cytokine networks to the regulatory machinery of melanogenesis. It provides novel perspectives for deciphering the pathogenesis of pigmentary skin disorders and developing therapeutic strategies with combined anti-inflammatory and depigmenting effects.

## 1 Introduction

Normal skin color is primarily determined by the pigment content within the skin and differences in skin anatomy, among which melanin is the main pigment responsible for skin color (1). Melanin plays physiological roles in protecting against ultraviolet radiation, antioxidation, and scavenging free radicals (2, 3). However, abnormal melanin accumulation can lead to skin hyperpigmentation, such as melasma, freckles, and post-inflammatory hyperpigmentation (4). When hyperpigmentation affects the face or other exposed areas, it often has a significant negative impact on patients’ self-esteem, confidence, and social interactions (5). A questionnaire survey revealed that up to 47.3% of patients with pigmented skin disorders experience psychological distress due to skin lesions (6). Nevertheless, the exact pathogenesis of skin hyperpigmentation remains unclear, and current preventive and therapeutic options are limited. Prevention mainly relies on daily protection from ultraviolet exposure. In terms of treatment, in addition to etiological interventions, topical depigmenting agents, laser therapy, or chemical peeling may be used (5, 7, 8). However, even with timely and effective treatment, skin hyperpigmentation tends to improve slowly, and treatment outcomes are often unsatisfactory. Therefore, in-depth elucidation of the regulatory mechanisms underlying melanogenesis and the identification of more effective intervention targets are not only a research hotspot in dermatology but also hold significant potential for clinical translation.

Melanogenesis is a complex biochemical process catalyzed by the tyrosinase family within melanocytes and is finely regulated by multiple signaling pathways, including α-melanocyte-stimulating hormone and stem cell factor (9, 10). Recent studies have revealed that the inflammatory microenvironment plays a critical role in melanin metabolic abnormalities. Multiple lines of evidence indicate that keratinocytes, fibroblasts, and vascular endothelial cells, upon exposure to ultraviolet radiation or mechanical stimulation, secrete various inflammatory mediators such as IL-1, IL-6, TNF-α, PGE2, and ET-1. These factors act on melanocytes in a paracrine manner and regulate signaling pathways including nuclear factor kappa B (NF-κB), PKA, and mitogen-activated protein kinase (MAPK), thereby influencing the activity and expression of tyrosinase and its related proteins (11, 12). Ultimately, these processes modulate melanogenesis and establish a feedback loop of “inflammation–melanin metabolic disorder.” Furthermore, under heat stress conditions, melanocytes can autocrine the inflammatory chemokine CX3CL1, which upregulates the expression of key factors such as MITF and TYR via activation of the JNK signaling pathway, thereby promoting melanogenesis (13). Therefore, linking the inflammatory cytokine network with the regulatory mechanisms of melanogenesis may provide new research clues for elucidating the pathogenesis of hyperpigmentary skin disorders and for developing therapeutic strategies that combine anti-inflammatory and depigmenting effects.

CXCL1 (CXC motif chemokine ligand 1) is an important member of the CXC chemokine family (14). By binding to and activating its G protein-coupled receptor CXCR2, CXCL1 exerts multiple biological functions, including chemotaxis of neutrophils, regulation of inflammatory responses, and promotion of angiogenesis (15, 16). Notably, the role of CXCL1 in regulating melanocyte function has recently gained increasing attention. Studies have shown that upon stimulation by melanogenic factors such as bFGF, ET-1, and α-MSH, normal human epidermal melanocytes release CXCL1, exogenous CXCL1 enhances the proliferative effects of these factors on melanocytes, whereas blockade of CXCR2 inhibits this effect, suggesting the existence of a CXCL1/CXCR2 autocrine growth loop in melanocytes (17). Furthermore, in inflammatory skin diseases such as psoriasis, IL-17 and TNF-α synergistically suppress the expression of melanogenesis-related genes while also inducing CXCL1 production in melanocytes (18). Collectively, these findings suggest that CXCL1 within the inflammatory microenvironment may participate in regulating the balance between melanocyte proliferation and melanogenic function. Our previous study, which established machine learning-based evaluation models for post-inflammatory pigmentation abnormalities, similarly found that CXCL1 exhibits high predictive value for predicting pigmentation outcomes following skin inflammation. In that study, we also preliminarily observed that CXCL1 reduced melanin content in MNT1 cells, suggesting that CXCL1 may negatively regulate melanogenesis (19). However, that study did not systematically investigate the precise role and underlying mechanisms of CXCL1 in melanogenesis. Overall, whether CXCL1 directly participates in the regulation of melanogenesis and its downstream signaling pathways have not yet been systematically reported. Therefore, the present study aims to investigate the role of CXCL1 in melanogenesis and its potential signaling pathways, hoping to provide new insights into the pathogenesis of hyperpigmentary skin disorders and to identify potential molecular targets for their clinical intervention.

## 2 Experimental section

### 2.1 Transcriptome data analysis

The RNA expression profile data used in this study were downloaded from the public database GEO (Gene Expression Omnibus, http://www.ncbi.nlm.nih.gov/geo/). The specific datasets included: the melasma skin tissue dataset GSE72140 (20), the UVB-irradiated human primary melanocyte dataset GSE70280 (21), the psoriasis skin tissue datasets (GSE13355 (22), GSE30999 (23), GSE41664 (24)), the acne skin tissue dataset GSE53759 (25), the atopic dermatitis skin tissue dataset GSE133477 (26), and the ultraviolet-irradiated human skin tissue dataset GSE45493 (27). First, in each dataset, we used single-sample gene set enrichment analysis (ssGSEA) to calculate a weighted score (termed mela-score) for a melanogenesis-related gene set (including PMEL, MLANA, TYR, MITF, DCT, WNT4, and WNT2B), and analyzed its correlation with CXCL1 expression levels. Subsequently, in the ultraviolet-irradiated human skin tissue dataset GSE45493, samples were divided into CXCL1 high-expression and low-expression groups based on the median CXCL1 expression level. The limma package (version 3.66.0) was used to identify differentially expressed genes (DEGs) between the two groups, with screening thresholds set at P < 0.05 and |log_2_FC| > 0.5. On this basis, Kyoto Encyclopedia of Genes and Genomes (KEGG) pathway enrichment analysis and gene set enrichment analysis (GSEA) were further performed using the clusterProfiler package (version 4.18.4). Data visualization was carried out using the ggplot2 package (version 4.0.3) and pheatmap package (version 1.0.13). All analyses were conducted using R (version 4.5.2) on Ubuntu 22.04 LTS. Detailed information for all datasets is provided in **Supplementary Material Table 1**.

### 2.2 Reagents

Recombinant human CXCL1 was obtained from PeproTech (USA). The BIFTYR probe was prepared by Xiangya School of Pharmaceutical Sciences, Central South University. The Fontana-Masson staining kit was purchased from G-ClONE (Beijing, China). Triton X-100 was acquired from Sigma-Aldrich, while Sodium deoxycholate and L-DOPA were supplied by Solarbio (Beijing, China). A 4% neutral paraformaldehyde solution and the Cell Counting Kit-8 (CCK-8) were sourced from Biosharp (Beijing, China). Culture media and supplements, including Dulbecco’s Modified Eagle Medium (DMEM), Medium 254 (#M-254-500), human melanocyte growth supplement (HMGS), penicillin/streptomycin (P/S), and penicillin/streptomycin/amphotericin B were obtained from Gibco (Maryland, USA). SB225002 (KM9998) was purchased from KKL Med Inc (USA). The primary antibodies used in this study, along with their respective suppliers, are listed as follows: GAPDH (#2118, Cell Signaling Technology), TYR (R381782, ZENBIO), MITF (R24980, ZENBIO), TYRP1 (R382326, ZENBIO), DCT (ER65221, HUABIO), PMEL17 (#H1219, Santa Cruz), β-catenin (51067-2-AP, Proteintech), and CXCL1 (P12654, ProMab Biotechnologies Inc.).

### 2.3 Cell culture

The human melanoma cell line MNT1 and the fetal bovine serum (FBS) required for its culture were purchased from Meisen (China, Zhejiang). The MNT1 cells were cultured in DMEM medium supplemented with 20% FBS and 1% P/S. In addition, with the approval of the Ethics Committee of the Third Xiangya Hospital, Central South University, foreskin tissues were collected from healthy volunteers (aged ≤ 30 years) undergoing circumcision in the Department of Urology of the same hospital. Human primary epidermal melanocytes (MCs) were subsequently isolated from these tissues. The melanocytes were cultured in Medium 254 supplemented with 1% HMGS and 1% penicillin/streptomycin/amphotericin B. All cells were cultured in a constant temperature cell culture incubator set at 37 °C with 5% CO_2_.

### 2.4 CCK-8 assay for cell proliferation activity

A 100μL cell suspension containing 5,000 cells was seeded into each well of a 96-well plate. After complete cell attachment, the supernatant was discarded, and the cells were treated with CXCL1 solutions at various concentrations (0, 10, 25, 50, 100, 200 ng/mL) for an additional 24h or 48h. Following the treatment, 10μL of CCK-8 solution was added to each well, followed by gentle mixing (all operations were performed under strict light protection). The plate was then incubated in a cell culture incubator in the dark for 2 hours. Subsequently, the absorbance at 450 nm (A450) was measured using a multimode microplate reader (PerkinElmer EnVision Xcite, UK).

### 2.5 Melanin content measurement

**Masson-Fontana staining and Dopa staining**: Cells were evenly seeded into 12-well plates. After reaching 30%–40% confluence, they were treated with various concentrations of CXCL1 solution for 48 hours. Subsequently, the cells were fixed with 4% paraformaldehyde solution at room temperature for 20–30 minutes, thoroughly rinsed with double-distilled water, and then incubated with ammoniacal silver solution in each well for approximately 30 minutes in a 56 °C water bath in the dark. After another rinse with double-distilled water, sodium thiosulfate solution was added to each well for 1–5 minutes. For dopa staining, following fixation, 0.1% L-DOPA solution was added to each well and incubated in a 37 °C water bath in the dark, with staining observed every 2 hours. For paraffin sections, after deparaffinization and hydration, the sections were incubated with ammoniacal silver solution at 56 °C in the dark for approximately 30 minutes. After rinsing with distilled water, the nuclei were counterstained with neutral red solution for 5 minutes. Finally, melanin staining was observed and photographed using an inverted fluorescence microscope (Zeiss, Axio Vert.A1, Germany).

**NaOH lysis method**: Cells were evenly seeded into culture dishes. After reaching over 50% confluence, they were treated with various concentrations of CXCL1 solution for 48 hours. A total of 1×10^6^ cells were collected into 1.5 mL EP tubes and centrifuged at 1000 rpm for 5 minutes, and the supernatant was discarded. Then, 1 mL of solution containing 10% DMSO (diluted in 1 mM NaOH) was added, and the cells were incubated in an 80 °C water bath for 1 hour to lyse the cells and dissolve the melanin. After incubation, the mixture was transferred to a 96-well plate. The absorbance at 490 nm (A_490_) was measured using a multimode microplate reader.

### 2.6 Tyrosinase activity assay

**BIFTYR probe method** (28): Cells were evenly seeded into 12-well plates. After reaching 30%–40% confluence, they were treated with various concentrations of CXCL1 solution for 48 hours. Subsequently, 20μM BIFTYR probe solution was added to each well, and the plates were incubated in a 37 °C incubator for 1 hour in the dark. After washing with pre-warmed PBS, fluorescence intensity was observed using an inverted fluorescence microscope to evaluate intracellular tyrosinase (TYR) activity.

**L-DOPA method**: Cells were evenly seeded into culture dishes. After reaching over 50% confluence, they were treated with various concentrations of CXCL1 solution for 48 hours. The cells were then collected, washed with PBS, and 1×10^6^ cells were resuspended in 0.5 mL of 0.5% sodium deoxycholate solution. The cells were lysed at 4 °C for 15 minutes and subsequently incubated in a 37 °C water bath for 10 minutes. Then, 1 mL of 0.1% L-DOPA solution was added to each tube. After mixing, the solution was transferred to a 96-well plate and allowed to react for 30 minutes at 37 °C in the dark. The absorbance at 475 nm (A_475_) was measured using a multimode microplate reader.

### 2.7 Quantitative real-time PCR

Total RNA was extracted using a rapid RNA extraction kit (FASTAGEN, Shanghai, China), and its concentration and purity were measured using a NanoDrop spectrophotometer. A total of 1μg of RNA was reverse-transcribed into cDNA (Vazyme, Nanjing, Chian). The qPCR reaction was performed using the qPCR SuperMix kit (Vazyme) on Roche LightCycler480II (Basel, Switzerland). The reaction conditions were as follows: initial denaturation at 95 °C for 30 seconds, followed by 40 cycles of denaturation at 95 °C for 10 seconds, annealing at 56 °C for 30 seconds, and extension at 72 °C for 30 seconds. GAPDH was used as an internal control, and the relative expression levels of target genes were calculated using the 2^ΔΔCt method. Primer sequences are provided in **Supplementary Material Table 2**.

### 2.8 Western blotting

Total protein was extracted using RIPA lysis buffer (supplemented with 10% protease inhibitor and 10% phosphatase inhibitor), and protein concentrations were determined using the BCA method. Equal amounts of protein (20–30μg) were separated by SDS-PAGE and then transferred onto PVDF membranes (0.22μm, Millipore, USA). The membranes were blocked with rapid blocking solution (Biosharp, Shanxi, China) for 10 minutes at room temperature and subsequently incubated with primary antibodies (diluted at 1:1000 or 1:2000) overnight at 4 °C. After washing with TBST, the membranes were incubated with HRP-conjugated secondary antibodies for 1 hour at room temperature. Immunoreactive signals were visualized using an enhanced chemiluminescence (ECL) method, and the fluorescence intensity on the PVDF membranes was detected using the Odyssey CLx imaging system (Li-COR Biosciences).

### 2.9 Immunofluorescence

Cells were evenly seeded into 12-well plates. After reaching 30%–40% confluence, they were treated with various concentrations of CXCL1 solution for 48 hours. Subsequently, the cells were fixed with 4% paraformaldehyde at room temperature for 20–30 minutes. Following fixation, the cells were permeabilized with 0.3% Triton X-100 solution (diluted in PBS) for 10 minutes at room temperature and then blocked with 5% BSA solution (diluted in PBS) for 1 hour. The cells were then incubated with primary antibody (β-catenin, diluted at 1:400) overnight at 4 °C. The next day, the cells were incubated with goat anti-rabbit fluorescent secondary antibody 488 (diluted at 1:400) for 1 hour at room temperature in the dark, followed by incubation with DAPI for 5 minutes in the dark. Finally, the fluorescence intensity of the cells was observed using an inverted fluorescence microscope.

### 2.10 Immunohistochemistry

Following approval from the patients and the Ethics Committee of the Third Xiangya Hospital, Central South University, foreskin tissue samples were collected from healthy volunteers (aged ≤ 30 years) undergoing circumcision. After removing the subcutaneous tissue and fat, the foreskin was cut into approximately 2 cm² pieces. The skin tissue pieces were then placed with the epidermal side facing upward in DMEM medium containing 10% FBS and 1% penicillin/streptomycin/amphotericin B, and cultured in an incubator at 37 °C with 5% CO_2_ for 5 days, during which they were treated with various concentrations of CXCL1. The treated foreskin tissues were subsequently entrusted to Servicebio (Wuhan, China) for paraffin embedding and sectioning. Paraffin sections were subjected to deparaffinization, hydration, antigen retrieval, endogenous peroxidase inactivation, and blocking, followed by incubation with primary antibody (β-catenin and CXCL1, diluted at 1:400) overnight at 4 °C. The next day, after incubation with reaction enhancement solution for 20 minutes and secondary antibody for 40 minutes, staining was performed using DAB (ZLI-9018, ZSGB-BIO). The stained sections were observed under an inverted fluorescence microscope.

## 3 Results

### 3.1 CXCL1 Is negatively correlated with melanogenesis

**Figure 1:**
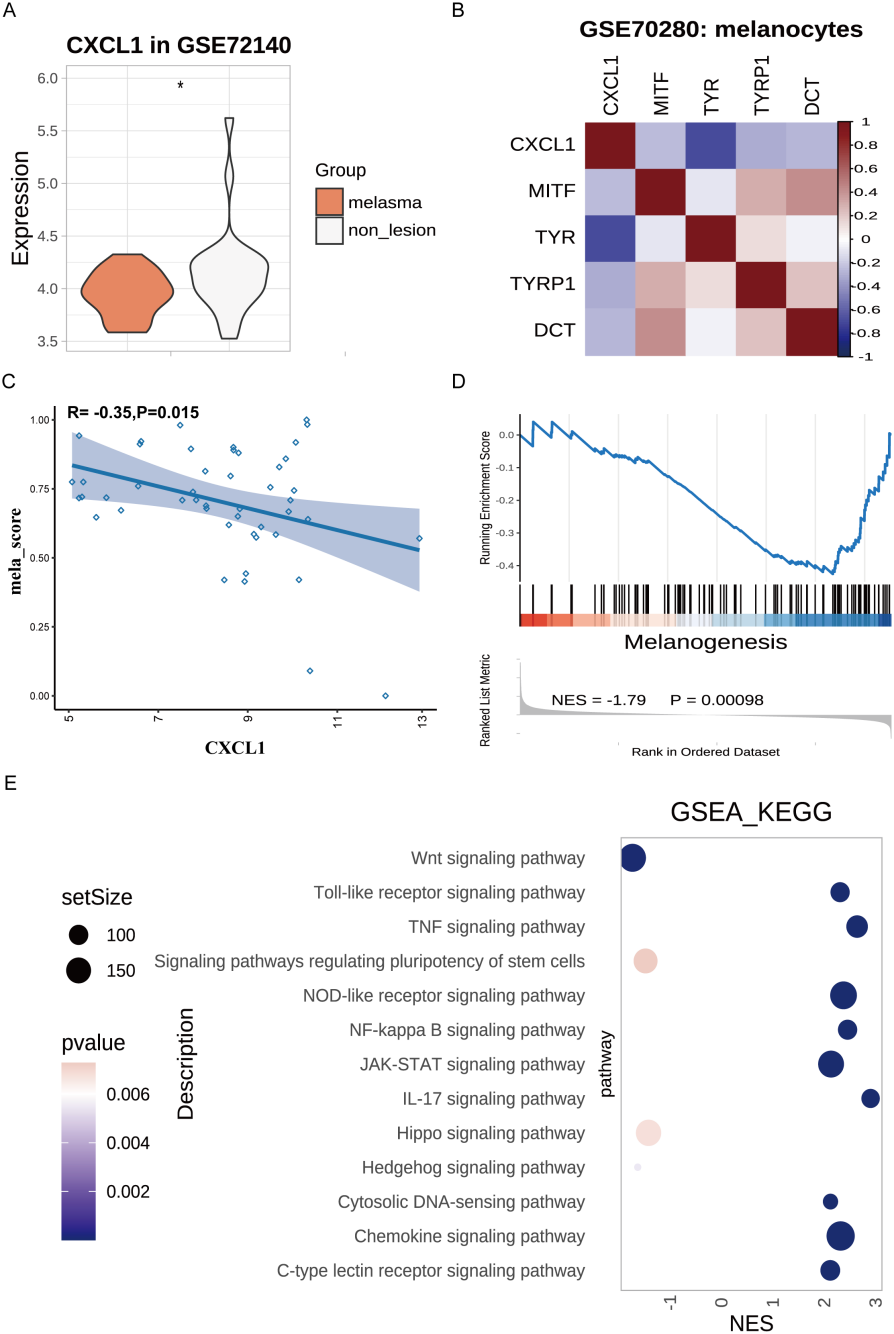
CXCL1 is negatively correlated with melanogenesis. **(A)** Violin plot showing the expression levels of CXCL1 in the melasma skin tissue transcriptomic dataset (GSE72140). **(B)** Correlation analysis between CXCL1 and key melanogenesis-related genes (MITF, TYR, TYRP1, DCT) in the UVB-irradiated human primary melanocyte transcriptomic dataset (GSE70280). **(C)** Correlation analysis between CXCL1 and the melanogenesis score (mela-score) in the ultraviolet-irradiated human skin tissue transcriptomic dataset (GSE45493). **(D, E)** GSEA results based on KEGG pathways in the GSE45493 dataset.

To investigate whether CXCL1 is involved in the regulation of melanogenesis, we analyzed the transcriptomic dataset of melasma skin tissue (GSE72140) and found that CXCL1 expression was significantly downregulated in melasma skin tissues (**Figure 1A**). Meanwhile, in the transcriptomic dataset of human melanocytes irradiated with melanogenesis-inducing doses of UVB (GSE70280), correlation analysis between CXCL1 and key melanogenesis-related genes (MITF, TYR, TYRP1, DCT) revealed that CXCL1 expression was significantly negatively correlated with these genes (**Figure 1B**). Consistently, in the transcriptomic dataset of human skin tissue irradiated with melanogenesis-inducing doses of ultraviolet radiation (GSE45493), as well as in datasets of psoriasis (GSE13355, GSE30999, GSE41664), acne (GSE53759), and atopic dermatitis (GSE133477), CXCL1 was also found to be significantly negatively correlated with the mela-score (a weighted score calculated by ssGSEA for the melanogenesis-related gene set including PMEL, MLANA, TYR, MITF, DCT, WNT4, and WNT2B) (**Figure 1C**; **Supplementary Figure 1**). These findings suggest that CXCL1 may be negatively associated with melanogenesis in various skin pigmentation-related disorders.

To further clarify the regulatory relationship between CXCL1 and melanogenesis, we divided the samples in GSE45493 into CXCL1 high-expression and low-expression groups based on the median CXCL1 expression level. Through analysis of differentially expressed genes (DEGs) between the two groups (**Supplementary Figures 2A, B**) and subsequent GSEA enrichment analysis based on the KEGG database, we found that the “melanogenesis” functional set was significantly enriched in the CXCL1 low-expression group (**Figure 1D**). Moreover, multiple signaling pathways that positively regulate melanogenesis, including the “WNT signaling pathway”, “Hippo signaling pathway”, and “Hedgehog signaling pathway” were significantly enriched in the CXCL1 low-expression group (**Figure 1E**). In addition, functional annotation of the top 200 DEGs (ranked by P-value) in the KEGG database also revealed significant enrichment of melanogenesis-related pathways such as the “WNT signaling pathway”, “MAPK signaling pathway”, and “Hippo signaling pathway” (**Supplementary Figure 2C**).

### 3.2 CXCL1 suppresses melanogenesis

**Figure 2:**
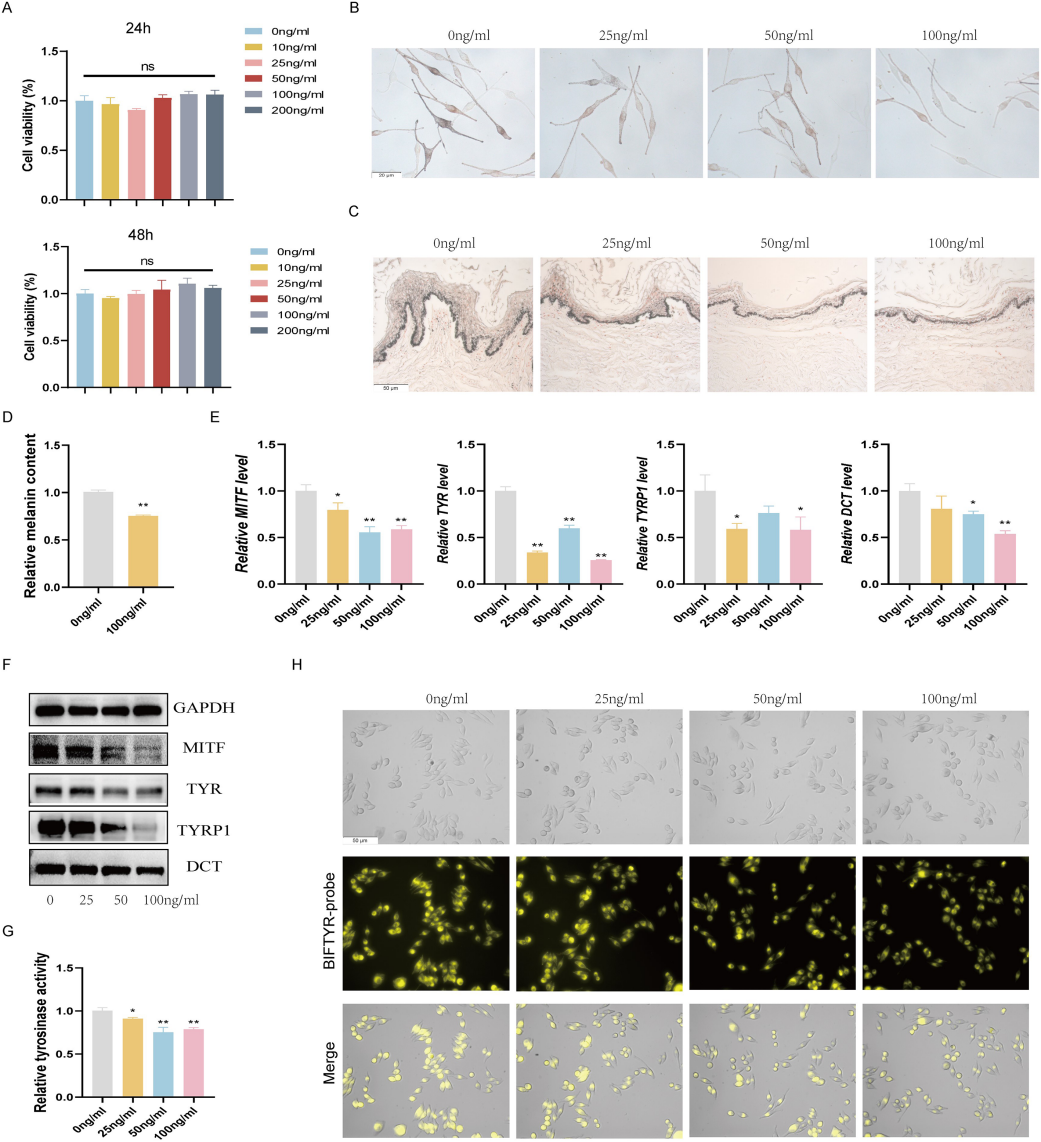
The CXCL1-CXCR2 axis negatively regulates melanogenesis by suppressing the WNT signaling pathway. **(A)** Cell viability was assessed using the CCK-8 assay in MNT1 cells treated with various concentrations of CXCL1 for 48 hours. **(B, C)** Melanin content was observed using Fontana-Masson staining in MCs treated with various concentrations of CXCL1 for 48 hours **(B)** and in human skin tissue treated for 5 days **(C)**. **(D–H)** After MNT1 cells treated with various concentrations of CXCL1 for 48 hours: Intracellular melanin content was quantified using the NaOH lysis method **(D)**; the expression levels of key melanogenesis-related genes (MITF, TYR, TYRP1 and DCT) were detected by qPCR **(E)** and Western blot **(F)**; and tyrosinase activity was detected using the L-DOPA method **(G)** and a BIFTYR fluorescent probe **(H)**. * indicates p < 0.05; ** indicates p < 0.01.

To validate the above bioinformatics findings and clarify the negative regulatory role of CXCL1 in melanogenesis, we exogenously treated human primary melanocytes (MCs), melanin-rich MNT1 cells (a tool cell line for melanogenesis research), and ex vivo human foreskin tissue with various concentrations of CXCL1. The results showed that CXCL1 had no cytotoxic effect at concentrations up to 200ng/mL (**Figure 2A**). Meanwhile, CXCL1 reduced melanin content in melanocytes and human skin tissue (**Figures 2B–D**; **Supplementary Figures 3A–C**). Furthermore, CXCL1 decreased the mRNA expression of key melanogenesis-related genes, including MITF, TYR, TYRP1, and DCT (**Figure 2E**), although the downregulation of TYR and TYRP1 was slightly attenuated at the concentration of 50 ng/ml. We further verified at the protein level by Western blot that CXCL1 markedly suppressed the expression of these key melanogenesis-related genes (**Figure 2F**). Consistent with these findings, tyrosinase activity, as a direct functional indicator of melanogenic capacity, was also significantly inhibited following CXCL1 treatment (**Figures 2G, H**). Collectively, these results demonstrate that CXCL1 exerts a negative regulatory effect on melanogenesis.

### 3.3 CXCL1 suppresses melanogenesis via its receptor CXCR2

**Figure 3:**
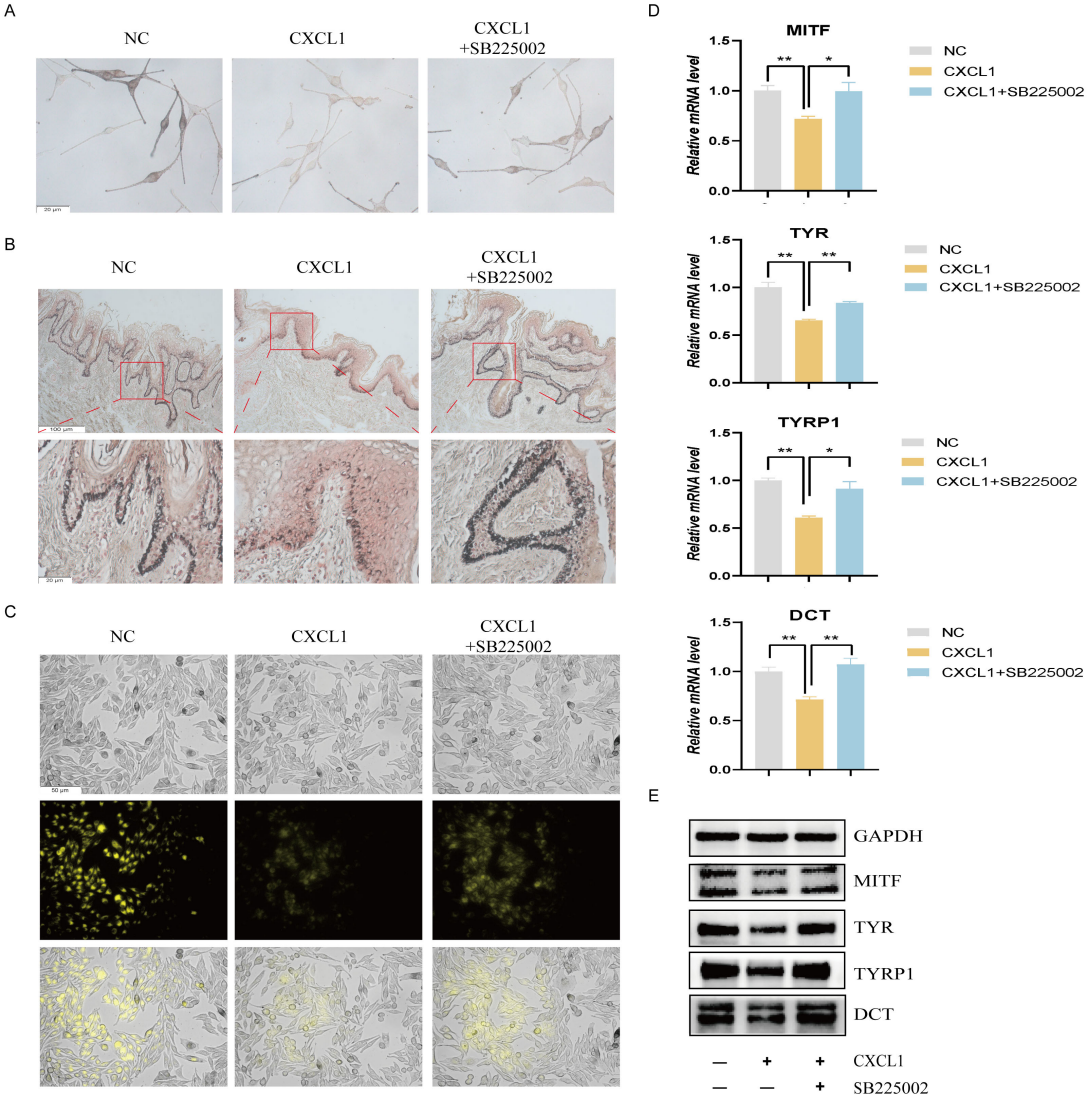
CXCL1 suppresses melanogenesis through its receptor CXCR2. **(A, B)** MCs **(A)** or human skin tissue **(B)** were pretreated with 5μM SB225002 for 30 minutes, followed by treatment with 100 ng/mL CXCL1 for 48 hours or 5 days, respectively. Intracellular melanin content was observed using Fontana-Masson staining. **(C–E)** MNT1 cells were pretreated with 5μM SB225002 for 30 minutes, followed by treatment with 100 ng/mL CXCL1 for 48 hours: Intracellular tyrosinase activity was detected using a BIFTYR fluorescent probe **(C)**; and the expression levels of key melanogenesis-related genes (MITF, TYR, TYRP1, DCT) were detected by qPCR **(D)** and Western blot **(E)**. * indicates p < 0.05; ** indicates p < 0.01.

CXCR2 is a key functional receptor for CXCL1. To investigate whether CXCL1 exerts its melanogenesis-inhibitory effects through CXCR2, we pretreated melanocytes and ex vivo human skin tissue with the specific CXCR2 inhibitor SB225002 (29), followed by exogenous CXCL1 treatment. The results showed that SB225002 effectively antagonized the CXCL1-induced reduction in melanin content (**Figures 3A, B**; **Supplementary Figure 4**) and tyrosinase activity (**Figure 3C**). Furthermore, the downregulation of key melanogenesis-related genes, including MITF, TYR, TYRP1, and DCT, induced by CXCL1 was also significantly reversed by SB225002 (**Figures 3D, E**). Collectively, these findings indicate that CXCL1 negatively regulates melanogenesis primarily through its receptor CXCR2.

### 3.4 CXCL1-CXCR2 axis suppresses melanogenesis by modulating the WNT signaling pathway

**Figure 4:**
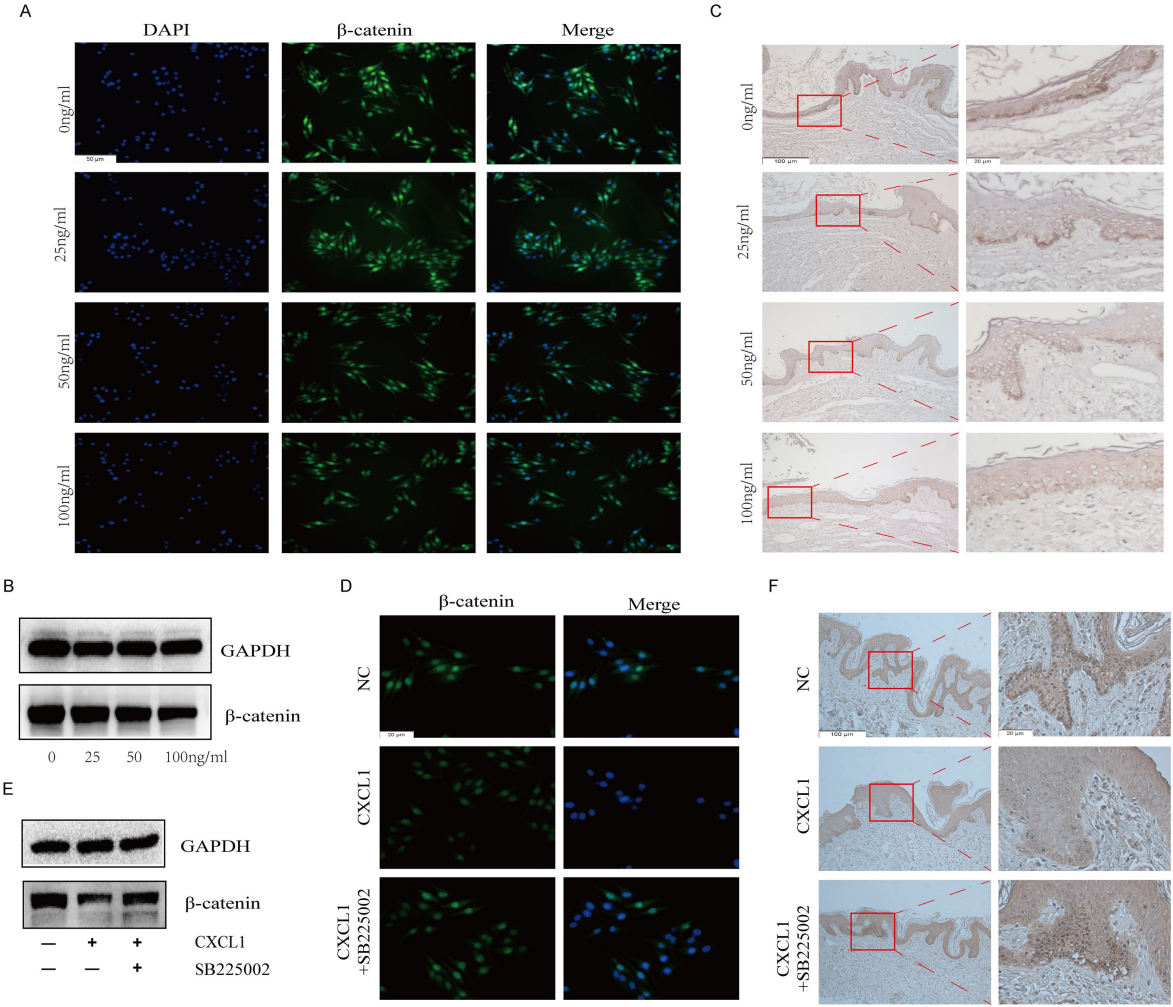
CXCL1 suppresses melanogenesis through its receptor CXCR2. **(A–C)** MNT1 cells were treated with various concentrations of CXCL1 for 48 hours, or human skin tissue was treated for 5 days. The distribution and expression of intracellular β-catenin were detected by IF **(A)**; the expression of intracellular β-catenin was detected by Western blot **(B)**; and the expression of β-catenin in skin tissue was detected by IHC **(C)**. **(D, E)** MNT1 cells or human skin tissue were pretreated with 5μM SB225002 for 30 minutes, followed by treatment with 100 ng/mL CXCL1 for 48 hours or 5 days, respectively. The distribution and expression of intracellular β-catenin were detected by IF **(D)**; the expression of intracellular β-catenin was detected by Western blot **(E)**; and the expression of β-catenin in skin tissue was detected by IHC **(F)**.

The WNT signaling pathway is one of the key pathways that promote melanogenesis (30). Our previous bioinformatics analysis revealed that the WNT signaling pathway was significantly enriched in the CXCL1 low-expression group in the GSE45493 dataset (**Figure 1E**). To further investigate whether the CXCL1-CXCR2 axis affects melanogenesis by regulating the WNT signaling pathway, we examined the expression of β-catenin, a key effector molecule of the WNT pathway (30), in CXCL1-treated MNT1 cells. Immunofluorescence results showed that CXCL1 treatment reduced both the nuclear translocation and the overall expression level of β-catenin (**Figure 4A**). This finding was consistent with the decreased total β-catenin protein expression detected by Western blotting (**Figure 4B**). Moreover, suppression of β-catenin expression was also observed in CXCL1-treated human skin tissue (**Figure 4C**). Subsequently, we further explored the role of CXCR2 in this process. The results demonstrated that the CXCR2 inhibitor SB225002 reversed the CXCL1-induced reduction in β-catenin expression both in MNT1 cells (**Figures 4D, E**) and in human skin tissue (**Figure 4F**).

## 4 Discussion

**Figure 5:**
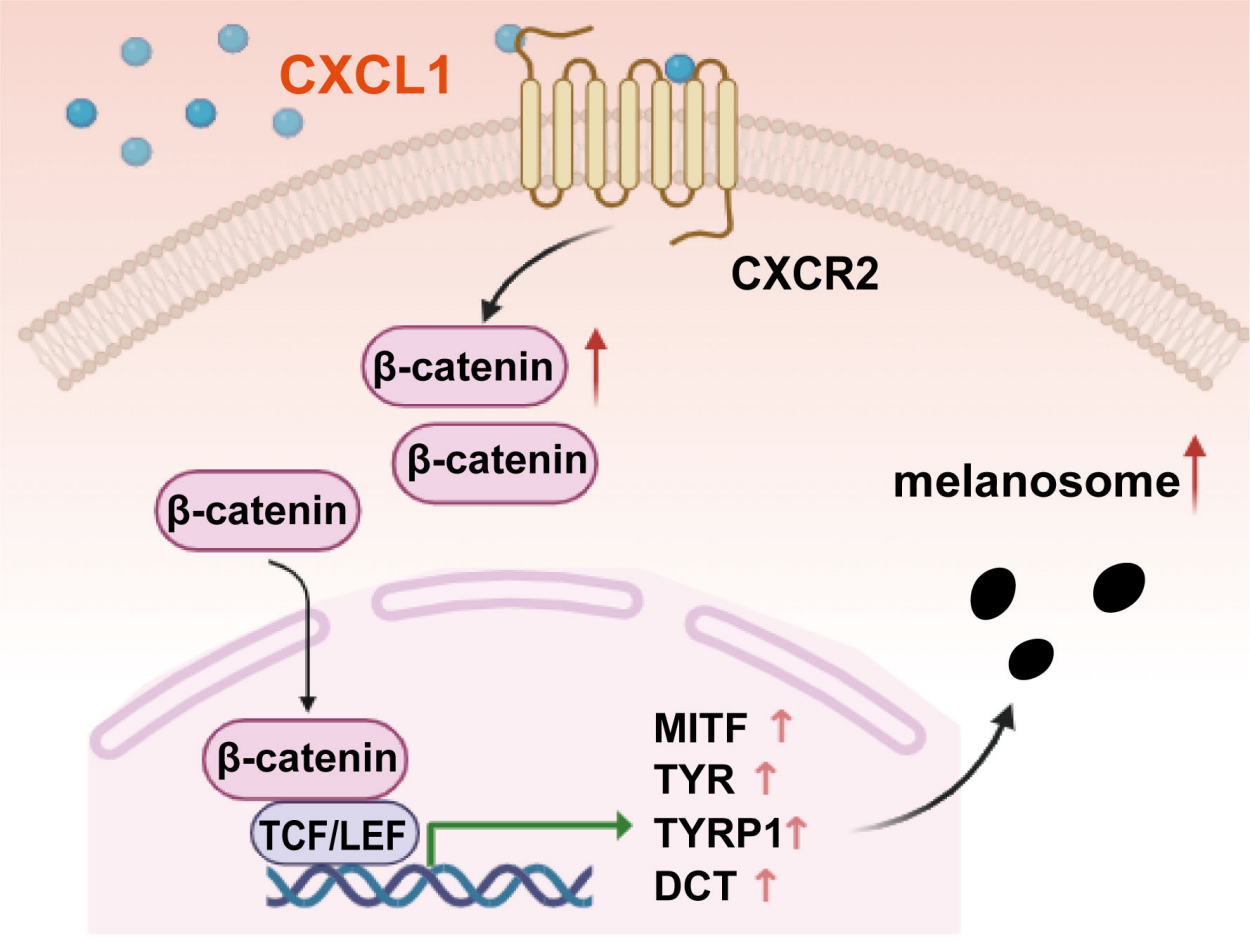
Mechanistic schematic of CXCL1-CXCR2 axis-mediated suppression of melanogenesis via WNT/β-catenin signaling.

By integrating public transcriptomic data with multi-level *in vitro* experimental validation, this study demonstrates that the CXCL1-CXCR2 axis suppresses melanogenesis through regulating the WNT/β-catenin signaling pathway (**Figure 5**).

Recent studies have consistently demonstrated that CXCL1 can be secreted and synthesized by various cell types within the dermis and epidermis, thereby participating extensively in skin repair and inflammatory pathological processes (31). During cutaneous wound healing, KLK14 secreted by keratinocytes induces fibroblasts to efficiently express and secrete CXCL1 through activation of the PAR-1 signaling pathway, which in turn positively regulates keratinocyte proliferation and migration, thereby promoting skin tissue repair and remodeling (32). Moreover, CXCL1 serves as a core regulator of cutaneous inflammatory responses and plays a critical role in the initiation and progression of various inflammatory skin diseases. It has been well established that CXCL1 recruits neutrophils to infiltrate lesional sites, amplifies local inflammatory cascades, and deeply participates in the pathogenesis of inflammatory skin disorders such as psoriasis and atopic dermatitis, rendering it an important molecular marker for assessing cutaneous inflammatory activity (31, 33). Furthermore, a limited number of studies have suggested that CXCL1 may be involved in the functional regulation of melanocytes (17, 18). However, these findings remain at the level of phenotypic association. Whether CXCL1 directly regulates melanogenesis, as well as its expression patterns and specific regulatory mechanisms in different pathological types of pigmentary skin disorders, has yet to be systematically elucidated.

Based on the above research background, we collected melasma, psoriasis, acne, atopic dermatitis, and UV-irradiated human skin tissue and melanocyte transcriptome datasets for analysis. The results revealed that CXCL1 expression levels were significantly negatively correlated with the melanogenesis score (mela-score) across multiple datasets, suggesting that CXCL1 may function as a negative regulator broadly involved in the regulation of skin hyperpigmentation under various pathological and stress conditions. Subsequently, by exogenously treating MC, MNT1, and ex vivo human skin tissue with CXCL1, we found that CXCL1 reduced melanin content, tyrosinase activity, and the expression of key melanogenesis-related genes, thereby confirming the negative regulatory role of CXCL1 in melanogenesis across multiple experimental dimensions. It should be noted that in the ex vivo human skin tissue culture model employed in this study, the penetration depth of exogenously applied recombinant proteins may be subject to physical constraints. Given the molecular size of CXCL1 (72amino acids, ~7.8kDa), further penetration into the deeper dermis may be limited by tissue barriers. Therefore, the CXCL1 expression and corresponding biological effects detected by IHC in this study primarily reflect the responses in the epidermal layer (particularly the stratum basale), while effects in the deeper dermis warrant further investigation.

CXCR2 is a specific functional receptor for CXCL1 and belongs to the classic G-protein-coupled receptor family. The specific binding of CXCL1 to CXCR2 initiates intracellular downstream multi-level signaling cascades, which serves as the core medium for CXCL1 to exert its biological functions (31, 34). To further clarify whether CXCL1 regulates melanogenesis in a CXCR2-dependent manner, we performed rescue experiments using SB225002, a specific inhibitor of CXCR2. The results showed that SB225002 significantly reversed the CXCL1-induced downregulation of melanin content, tyrosinase activity, and the expression of key melanogenic genes, confirming that the inhibitory effect of CXCL1 on melanogenesis is mediated by the CXCR2 receptor.

Among the multiple signaling pathways associated with pigment metabolism, the WNT/β-catenin pathway is a core classic pathway governing cutaneous melanogenesis, and its activity directly determines the melanin synthesis capacity of melanocytes. Activation of the WNT/β-catenin pathway stabilizes intracellular β-catenin and promotes its nuclear translocation. By binding to LEF/TCF transcription factors, it upregulates the expression of MITF, the core melanogenic transcription factor, and further activates downstream functional genes including TYR, TYRP1, and DCT, ultimately driving excessive melanin synthesis and abnormal pigment deposition (30, 35). Accumulating studies have demonstrated extensive crosstalk between CXCR2 and the WNT/β-catenin signaling pathway. The activation status of CXCR2 directly modulates the transcriptional activity and nuclear accumulation of β-catenin, and their interaction serves as a vital molecular mechanism regulating multiple cellular biological functions (36, 37). Notably, distinct from the previously reported canonical positive regulation of the WNT pathway by CXCR2 in tumors and stem cells, our bioinformatics analysis revealed significant enrichment of the WNT pathway in CXCL1-low expression samples, indicating a unique inverse regulatory correlation between CXCL1 and the WNT pathway in the cutaneous melanogenic microenvironment. Further experimental validation demonstrated that exogenous CXCL1 treatment markedly reduced the expression and nuclear translocation of β-catenin in MNT1 cells and ex vivo human skin tissues. Moreover, inhibition of CXCR2 by SB225002 effectively reversed the CXCL1-induced downregulation of β-catenin expression. Collectively, these findings indicate that the CXCL1-CXCR2 axis suppresses melanogenesis via inhibiting the WNT/β-catenin signaling pathway.

Notably, a well-documented crosstalk exists between BRAF signaling and the Wnt/β-catenin pathway. In normal human melanocytes, BRAF^V600E^ mutation enhances Wnt/β-catenin signaling activity (38), whereas in melanoma, BRAF^V600E^-mediated signaling suppresses Wnt/β-catenin activity, with β-catenin identified as a key regulator of BRAF^V600E^ -driven melanoma metastasis (38, 39). Furthermore, multiple studies have directly established a link between BRAF and CXCL1. In thyroid cancer, BRAF^V600E^ mutation promotes CXCL1 upregulation through the TBX3 regulatory pathway (40), and in colorectal cancer, BRAF^V600E^-mutant cell lines exhibit significantly higher basal CXCL1 expression than wild-type cells (41). In addition, CXCL1 overexpression markedly elevated the phosphorylation of BRAF at Ser446 (42), suggesting a bidirectional regulatory relationship between the two. Based on these findings, BRAF mutation status may modulate cellular fimmu.2026.1907373responsiveness to CXCL1 by altering the basal activity of the Wnt/β-catenin pathway, although this possibility warrants further investigation. Given that BRAF^V600E^ is the most common genetic alteration in melanoma (43), future exploration of CXCL1 function in the context of BRAF mutations may provide new insights into its biological roles across distinct genetic backgrounds.

Of note, bioinformatics analysis in the present study revealed that, in addition to the core WNT signaling pathway, other signaling pathways such as Hippo, MAPK, and Hedgehog were also significantly enriched in CXCL1 low-expression samples. The Hippo signaling pathway primarily regulates skin cell proliferation and differentiation through its downstream effector molecules YAP/TAZ. Moreover, it can respond to UVB-mediated METTL3 epigenetic modification and further regulate MITF transcriptional levels and melanocyte function by modulating YAP activity, thereby participating in UVB-induced melanogenesis (44). The MAPK signaling pathway is a classical signal transduction pathway in melanocytes, which primarily regulates MITF stability and transcriptional activity through protein phosphorylation modification, serving as a core pathway mediating melanin synthesis (45). The Hedgehog signaling pathway is involved in skin development and homeostasis maintenance and can also mediate heat stress-induced pigmentation. Heat stimulation can activate the keratinocyte TRPV3/Ca^2+^ pathway to initiate Hh signaling activation, which in turn positively promotes melanogenesis through epidermal paracrine effects (46). The enrichment of these multiple pathways suggests that the regulation of melanogenesis by the CXCL1-CXCR2 axis is not dependent solely on the WNT pathway but rather forms a complex molecular regulatory network by integrating multiple signaling pathways, synergistically achieving fine-tuned control of melanin synthesis. The present study focused exclusively on elucidating the core mechanism by which the CXCL1-CXCR2 axis regulates melanogenesis through the WNT pathway. However, the crosstalk patterns among these pathways, their upstream and downstream regulatory relationships, and the specific synergistic mechanisms remain incompletely understood. This multi-pathway regulatory hypothesis warrants further validation and refinement through deeper molecular experiments in future studies.

In conclusion, this study reveals a preliminary mechanism by which the CXCL1-CXCR2 axis negatively regulates melanogenesis through suppressing the WNT/β-catenin signaling pathway. This finding enriches the current understanding of melanin metabolic regulation within the inflammatory microenvironment and also provides a potential molecular target for the prevention and treatment of pigmentary skin disorders.

## Data Availability

The datasets presented in this study can be found in online repositories. The names of the repository/repositories and accession number(s) can be found in the article/**Supplementary Material**.
