# Supplementary material for: CXCL1-CXCR2 axis inhibits melanogenesis through the suppression of the WNT/β-catenin pathway
Source: Front Immunol. 2026 Jul 31;17:1907373. doi: 10.3389/fimmu.2026.1907373 (PMC13472829; doi:10.3389/fimmu.2026.1907373)
Supplement: Supplementary file 1 [file DataSheet1.pdf]

Supplementary Material

1 Supplementary Figures and Tables

1.1 Supplementary Tables

Supplementary Table 1. Information of GEO datasets:

GEO accession	Platform	Size	Source tissue	Experiment type
GSE72140	GPL570	48	Human skin	Array
GSE13355	GPL570	180	Human skin	Array
GSE30999	GPL570	170	Human skin	Array
GSE41664	GPL570	157	Human skin	Array
GSE133477	GPL570	242	Human skin	Array
GSE70280	GPL14550	24	Human melanocytes	
GSE45493	GPL6480	47	Human skin	Array

Supplementary Table 2. Information of primer sequences:

Gene	Forward	Reverse
GAPDH	CTCTGCTCCTCCTGTTCGAC	GCCCAATACGACCAAATCC
MITF	AAATACGTTGCCTGTCTCGG	TGTTGGGAAGGTTGGCTGGA
TYR	TCAGCCCAGCATCATTCTTC	GGCATCCGCTATCCCAGTAA
TYRP1	ACCAGAGGGTTCTCATAGTCAG	TTCTCAAATTGTGGCGTGTT
DCT	GGGCAGCGAGACCAGACGAT	TTGGCAATTTCATGCTGTTTCTTC

1.2 Supplementary Figures

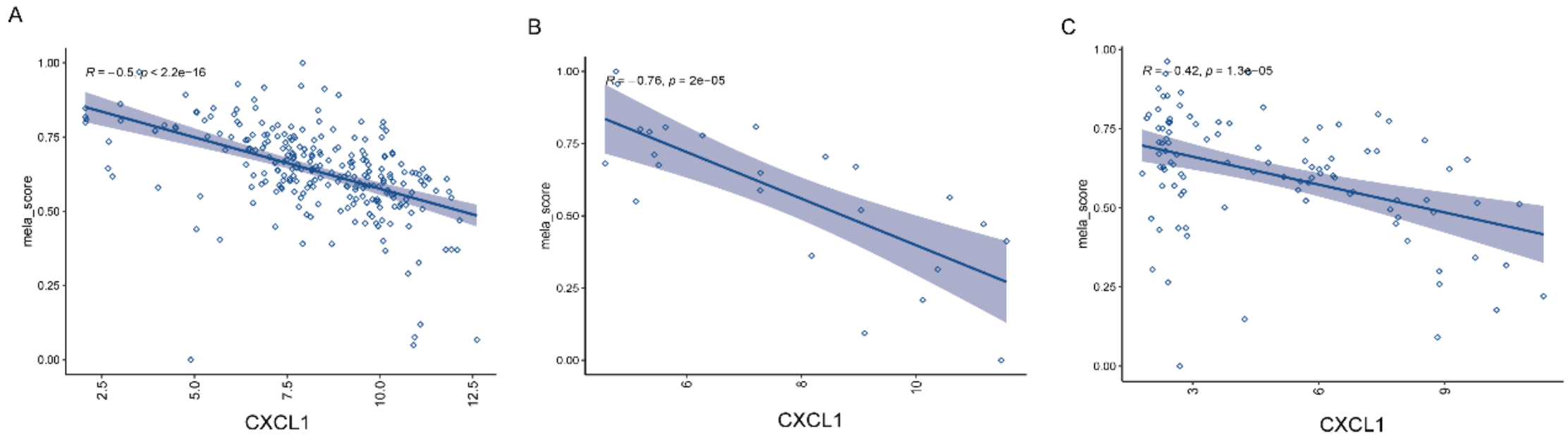

Supplementary Figure 1. (A-C) Correlation analysis between CXCL1 and the melanogenesis score (mela-score) in the psoriasis dataset which was generated by merging GSE13355, GSE30999, and GSE41664 (A), as well as in the acne dataset GSE53759 (B) and the atopic dermatitis dataset GSE133477 (C).

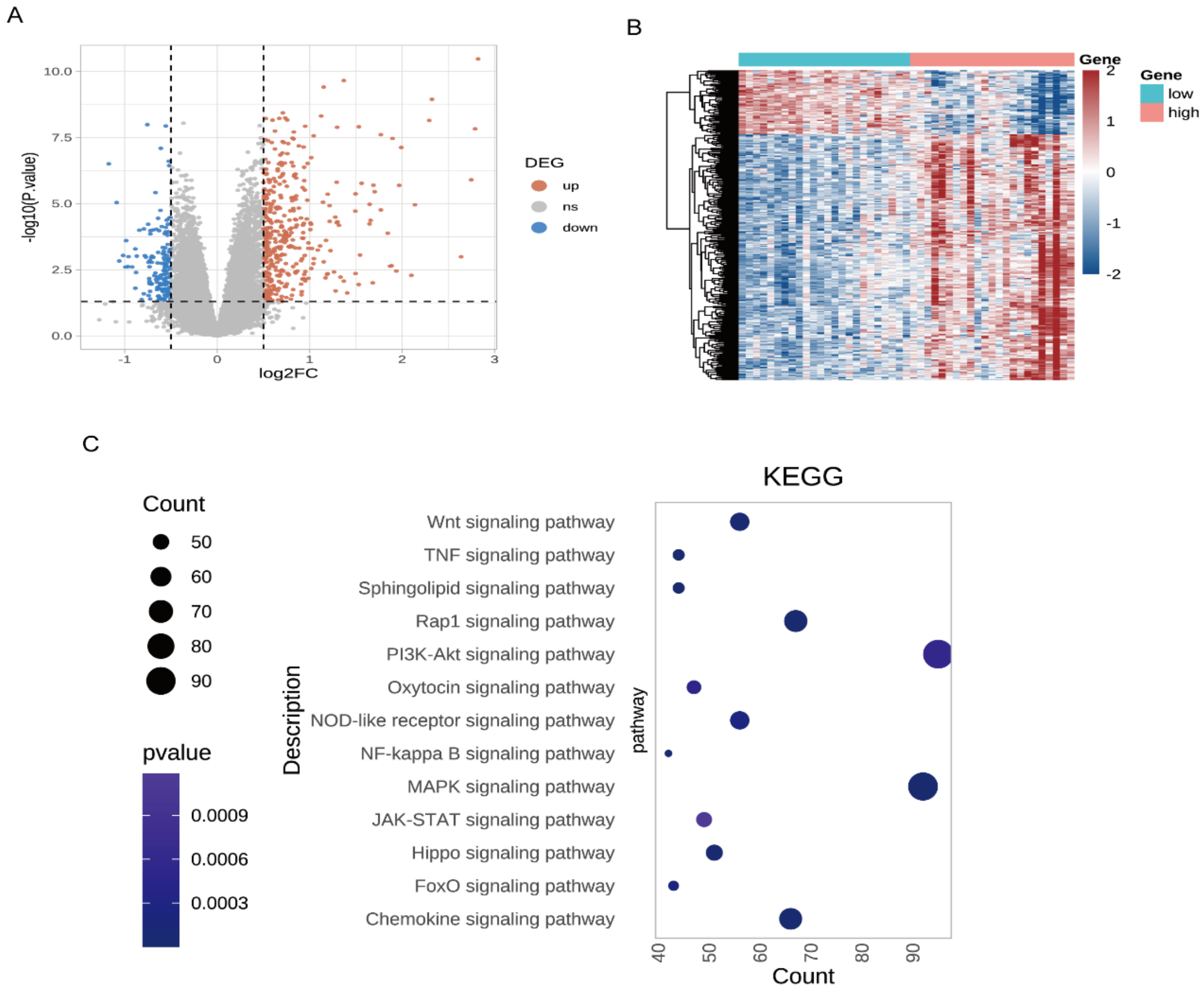

Supplementary Figure 2. (A-B) Visualization of DEGs grouped by CXCL1 expression in the GSE45493 dataset using a volcano plot (A) and a heatmap (B). (C) KEGG results in the GSE45493 dataset.

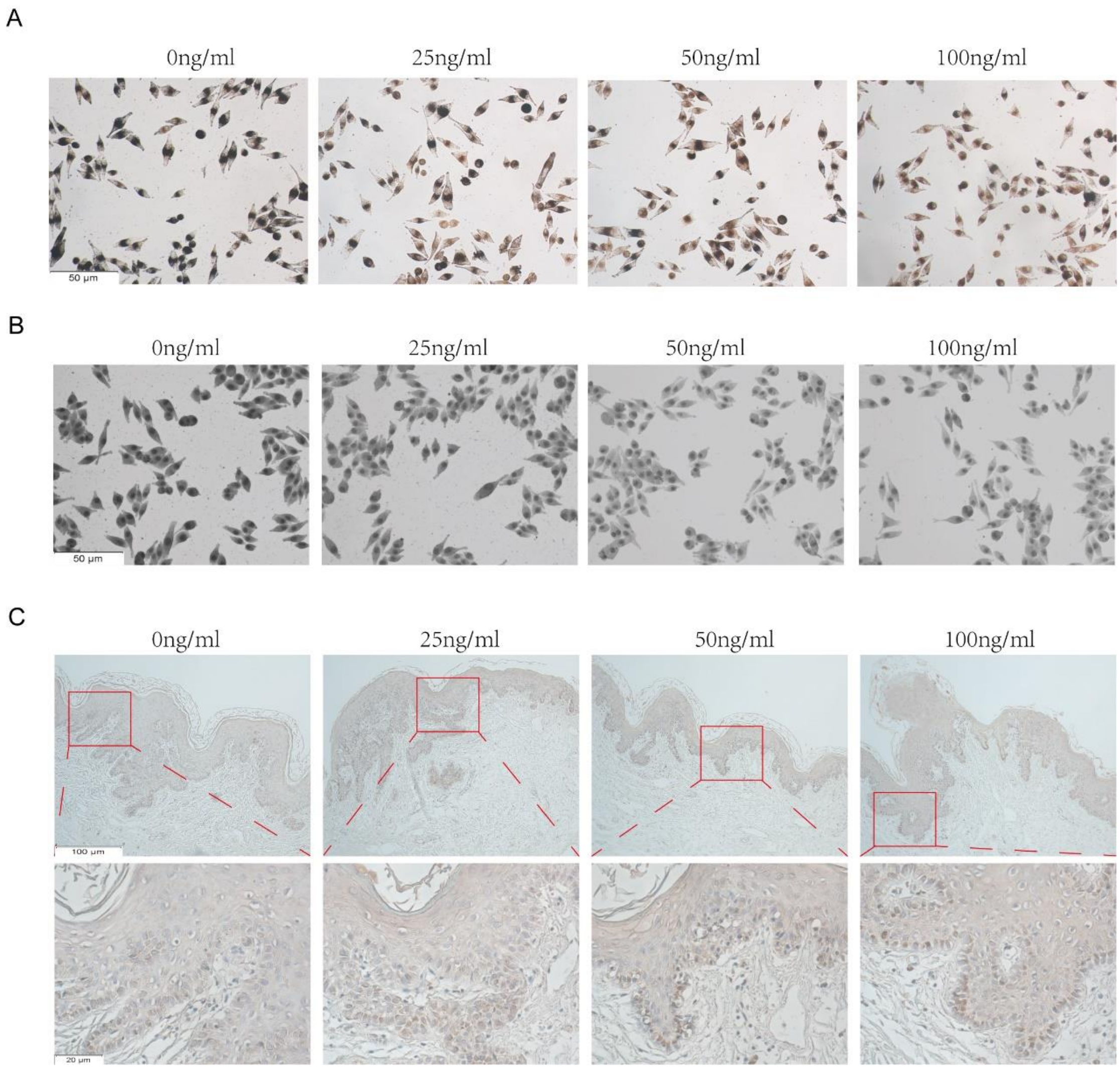

Supplementary Figure 3. MNT1 cells were treated with various concentrations of CXCL1 for 48 hours: Fontana-Masson staining (A) and Dopa staining (B) were used to detect the melanin content. (C) Human skin tissues were treated with various concentrations of CXCL1 for 5 days, and CXCL1 expression was examined by IHC.

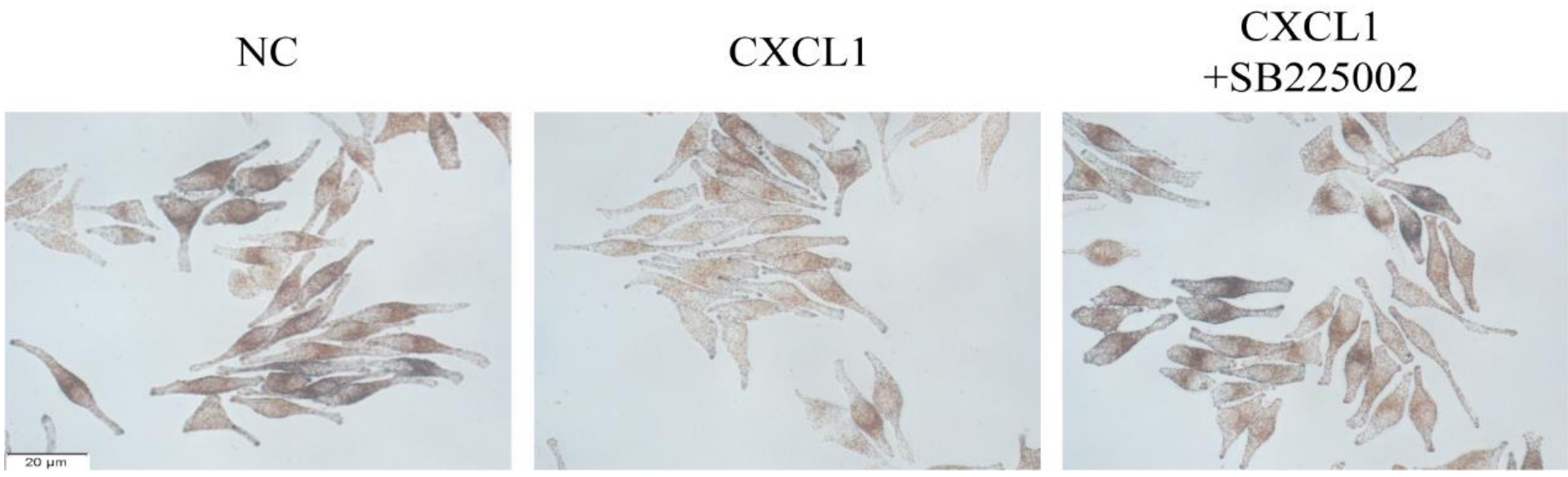

Supplementary Figure 4. (A) MNT1 cells were pretreated with 5μM SB225002 for 30 minutes, followed by treatment with 100 ng/mL CXCL1 for 48 hours. Intracellular melanin content was observed using Fontana-Masson staining.
